# Prenatal Vitamins and the Risk of Offspring Autism Spectrum Disorder: Systematic Review and Meta-Analysis

**DOI:** 10.3390/nu13082558

**Published:** 2021-07-26

**Authors:** Catherine Friel, Alastair H. Leyland, Jana J. Anderson, Alexandra Havdahl, Tiril Borge, Michal Shimonovich, Ruth Dundas

**Affiliations:** 1 Medical Research Council/Chief Science Office Social and Public Health Sciences Unit, University of Glasgow, Glasgow G3 7HR, UK; alastair.leyland@glasgow.ac.uk (A.H.L.); 2405470s@student.gla.ac.uk (M.S.); ruth.dundas@glasgow.ac.uk (R.D.); 2 Public Health Research Group, Institute of Health & Wellbeing, University of Glasgow, Glasgow G12 8RZ, UK; jana.anderson@glasgow.ac.uk; 3 Department of Mental Disorders, Norwegian Institute of Public Health, 222 Skoyen, 0213 Oslo, Norway; alexandra.havdahl@psykologi.uio.no; 4 Nic Waals Institute, Lovisenberg Diaconal Hospital, Postboks 4970 Nydalen, 0440 Oslo, Norway; 5 Promenta Research Center, Department of Psychology, University of Oslo, Boks 1072 Blindern, 0316 Oslo, Norway; 6 Division for Health Services, Cluster of Reviews and Health Technology Assessments, Norwegian Institute of Public Health, P.O. Box 222 Skoyen, 0213 Oslo, Norway; tiril.cecilie.borge@fhi.no

**Keywords:** autism spectrum disorder, folic acid, maternal nutrition, meta-analysis, multivitamin, systematic review

## Abstract

Prenatal nutrition is associated with offspring autism spectrum disorder (herein referred to as autism), yet, it remains unknown if the association is causal. Triangulation may improve causal inference by integrating the results of conventional multivariate regression with several alternative approaches that have unrelated sources of bias. We systematically reviewed the literature on the relationship between prenatal multivitamin supplements and offspring autism, and evidence for the causal approaches applied. Six databases were searched up to 8 June 2020, by which time we had screened 1309 titles/abstracts, and retained 12 articles. Quality assessment was guided using Newcastle–Ottawa in individual studies, and the Grading of Recommendations Assessment, Development and Evaluation (GRADE) for the body of evidence. The effect estimates from multivariate regression were meta-analysed in a random effects model and causal approaches were narratively synthesised. The meta-analysis of prenatal multivitamin supplements involved 904,947 children (8159 cases), and in the overall analysis showed no robust association with offspring autism; however, a reduced risk was observed in the subgroup of high-quality observational studies (RR 0.77, 95% CI (0.62, 0.96), I^2^ = 62.4%), early pregnancy (RR 0.76, 95% CI (0.58; 0.99), I^2^ = 79.8%) and prospective studies (RR 0.69, 95% CI (0.48, 1.00), I^2^ = 95.9%). The quality of evidence was very low, and triangulation was of limited utility because alternative methods were used infrequently and often not robustly applied.

## 1. Introduction

Autism spectrum disorder (hereafter “autism”) is a neurodevelopmental condition characterised by early-onset impairment in social communication and restricted and repetitive behaviour [1]. Prenatal nutrition may be a modifiable risk factor, which creates a potential target for prevention strategies and may reduce the significant public health implications of this condition. Autism in the U.K. is estimated to cost GBP 27 billion (EUR 29.8 billion) annually for health, education, and social care and in lost productivity [2] despite a modest prevalence of around 1.5% [3].

Previous reviews and meta-analysis reported a reduced risk of offspring autism associated with prenatal folic acid (FA) or multivitamin supplements [4,5], but as the conventional rhetoric states: “correlation does not imply causation”. The conditions necessary for estimating causality are greatly debated, and although randomised controlled trials are considered to be the gold-standard, their utility in nutritional epidemiology is limited because of ethical, financial, and practical barriers. For example, as the prevalence of autism is only 1.5%, a large sample size is necessary for adequate statistical power, but acquiring one is financially burdensome [6]. Conversely, a causal inference from non-experimental studies is problematic, largely due to bias [7]. For example, individuals may overreport compliance with prenatal nutritional supplements, creating a misclassification bias, or confounders, which influence both the exposure and outcome, create a spurious association.

Aetiological triangulation recognises that all approaches have bias and then exploits unrelated sources of bias to “test” the consistency of results [7]. Causal inferences are strengthened if we have consistent results across multiple approaches with different sources of bias, as the biasmay vary across different study designs, methods or analytical approaches. Some examples include conventional multivariate regression, gene-nutrient interactions, discordant sibling studies, cross-context comparisons and negative controls (see Lawlor et al. for an overview of approaches in triangulation [7]). We used the term “alternative causal approaches” to refer to the abovementioned approaches that are alternatives to conventional multivariate regression.

Evidence from alternative causal approaches is unsystematically synthesised in previous reviews, if at all, which limits transparency. As alternative causal approaches are increasingly applied within studies and may significantly alter causal reasoning, this evidence should be integrated in an explicit and scientifically rigorous process. This aligns with guidance from Cochrane [8] and a recent guideline on systematic aetiological reviews of observational studies [9]. We first reviewed the overall evidence from studies using conventional multivariate regression to investigate the association between prenatal nutritional status and autism in offspring. Second, we narratively synthesised the causal approaches. Lastly, we updated the search and addressed the limitations of previous reviews such as double-counting individual studies [5,10], or using the DerSimonian and Laird estimator, which can underestimate uncertainty [4,5].

## 2. Materials and Methods

The Preferred Reporting Items for Systematic Reviews and Meta-Analyses (PRISMA) were followed [11] (Supplementary Table S1). The review protocol was registered on the Prospective Register of Systematic Reviews, registration number: CRD42019154613. Available online: https://www.crd.york.ac.uk/prospero (accessed on 23 July 2021). 

### 2.1. Inclusion and Exclusion Criteria

The study designs were trials, cohort, case-controls and cross-sectional studies of any duration. We focused on the use of folic acid and multivitamin supplements (hereafter simply “multivitamin supplement”) in women during preconception and prenatal periods because folic acid is generally sourced from a multivitamin supplement [4]. Comparators were high versus low or no supplement intake. The outcome was offspring autism diagnosis based on the Diagnostic and Statistical Manual of Mental Disorders, International Classification of Disease, and health registers. There were no date limitations, but non-English language and animal studies were excluded.

### 2.2. Study Identification and Selection

The search strategy and selection of databases were guided by an information scientist with expertise in systematic reviews. Search strategies were adapted to each database. The following databases were searched from the earliest date to 8 June 2020; MEDLINE (OVID), EMBASE (OVID), PsycINFO (EBSCO), Web of Science core collection, Open Grey and BioRix. See Table 1 for MEDLINE search strategy.

Titles and abstracts were screened, full articles reviewed, and quality assessment was completed twice independently for each study, by C.F., T.B., and M.S. Disagreements were resolved through discussion and adjudicated, by J.A. Data were extracted by C.F. using a standardised form comprised of, author, year of publication, country and cohort, study design, sample size, age of participants, nutritional supplement, measure of autism, covariates, results, and causal approach.

### 2.3. Quality Assessment

The Newcastle–Ottawa Scale guided the quality assessment of each observational study. Scores range from 0 to 9, where a score of 7–9 was considered high quality in the subgroup analysis and consistent with similar previous reviews [5,12]. The Grading of Recommendations Assessment, Development and Evaluation (GRADE) approach was used to rate the body of evidence based on the degree of certainty in the result [8].

### 2.4. Data Synthesis and Analysis

We narratively synthesised studies that were inappropriate to meta-analysis. Additionally, we summarised the alternative approaches applied in table format.

### 2.5. Meta-Analysis

We conducted a meta-analysis of the fully adjusted effect estimates in a random effects model using the Hartung–Knapp–Sidik–Jonkman estimator [8]. Analyses of nutritional supplements were pooled if the exposure was categorical: no or low supplement intake as the reference category compared against supplement use. Autism is a rare outcome, so we assumed the odds ratio (OR) and hazard ratio (HR) were directly comparable to the relative risk (RR) [13]. The heterogeneity was measured with the Cochrane’s q and I^2^ statistics. The interpretation of heterogeneity (I^2^) was guided by Cochrane’s reference ranges [8]. Prediction intervals estimated the range of effect estimates that may be expected in individual settings that could improve the application of the research findings. These are distinct from the summary effect and 95% confidence intervals (CIs) that estimate the average effect of the exposure [8,14,15]. The R version 3.6.3 packages used were “meta” and “forestplots” [16]. Statistical tests of significance were 2-sided with an α of 0.05.

### 2.6. Sensitivity Analysis

Sources of heterogeneity were explored through the identification of outliers, leave-one-out analysis and subgroup analysis if there were ≥10 studies. A random effects model was used to estimate between and within subgroup effects. The pre-defined subgroups were study quality, study design (prospective/retrospective), region, mandatory fortification (yes/no), and stage of pregnancy, which was defined as the first trimester, compared against any point in pregnancy, if the nutritional supplement exposure period was undefined. Between-subgroup differences were measured with the q statistic and significance was indicated by *p* < 0.1 (2-sided). Small-study publication bias was assessed through the inspection of the funnel plot and Egger’s test [17]. The widely used DerSimonian and Laird estimator may underestimate uncertainty [8], but to facilitate comparison with previous research, we applied it in sensitivity analyses.

## 3. Results

### 3.1. Identification of Studies

**Figure 1:**
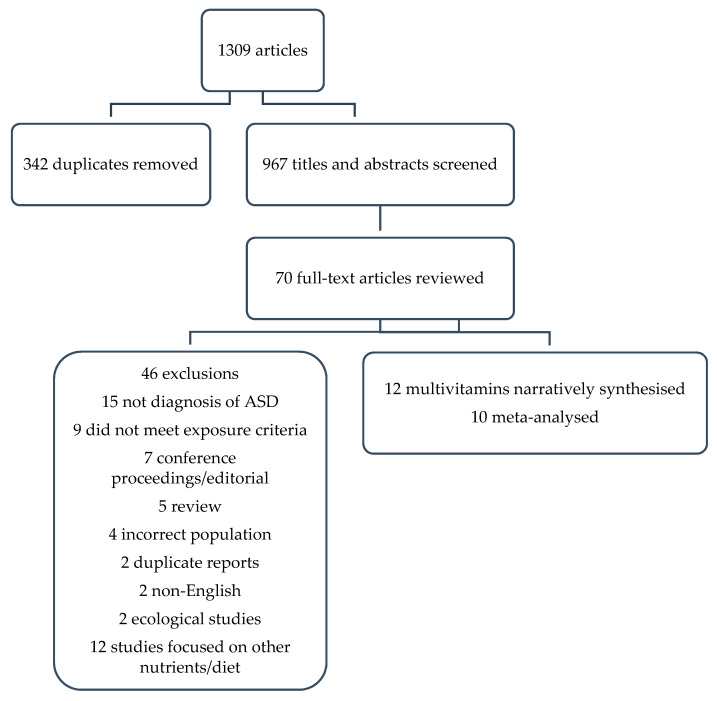
PRISMA flow chart of study selection.

A total of 1309 titles were identified with 342 duplicates, leaving 967 titles and abstracts to be screened (Figure 1). Of these, 897 were excluded based on title and abstract review, leaving 70 for a full-text review, of which 13 met the inclusion criteria. However, two reports were duplicated [18,19], and so the larger cohort was retained [19] leaving 12 studies in the final review, 10 of which were meta-analysed. The other two were narratively synthesised because the reference category was not low or no supplement use [20,21].

### 3.2. Quality Assessment

Based on the Newcastle–Ottawa Scale, five [20,22,23,24,25] of seven cohort studies [19,20,22,23,24,25,26] were of high quality, and two [27,28] of five case-control studies [21,27,28,29,30] were of high quality (Supplementary Tables S2 and S3). The quality of the body of evidence based on GRADE was very low (Table 2). The details for the evidence profile and rationale for GRADE rating are provided in Supplementary Tables S4 and S5. For a summary of studies and results, see Table 3.

### 3.3. Meta-Analylic Results

A total of 904,947 children including 8159 cases from six countries were included in the meta-analysis. All studies measured nutritional supplements and one included fortified food. The multivitamin dose was seldom reported.

**Figure 2:**
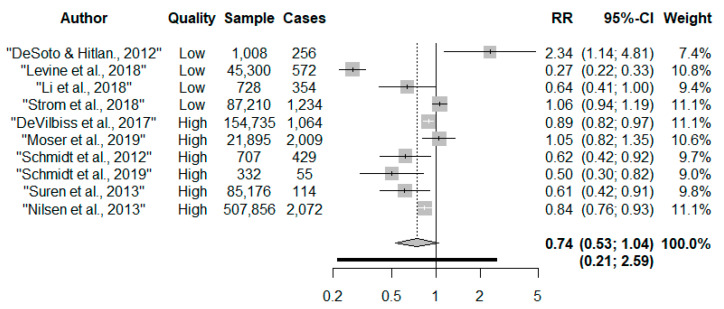
Forest plot of maternal multivitamin supplements and risk of offspring autism [19,21,22,23,24,25,26,27,28,30].

Overall, there was no robust evidence to associate taking prenatal multivitamins with autism risk compared to no/low intakes (RR 0.74, 95% CI: 0.53, 1.04) (Figure 2). The confidence intervals (CI) were wide, and there was considerable heterogeneity (I^2^ = 94.3%, *p* < 0.001). Egger’s test (*p* = 0.44) and inspection of the funnel plot suggests no evidence of asymmetry (Supplementary Figure S1). The precision increased when the DerSimonian and Laird estimator was applied compared to the Hartung–Knapp–Sidik–Jonkman estimator (RR 0.74, 95% CI: 0.57, 0.95, I^2^ = 94.3%). Upon removal of the outlier, DeSoto and Hitlan, there was a 32% reduced risk of autism (RR 0.68, 95% CI: 0.51, 0.91, I^2^ = 94.7%, *p* < 0.001); although heterogeneity remained considerable. There were no other influential studies (Supplementary Table S7). The 95% prediction interval indicated that the dispersion in the distribution of effect estimates was large and ranged from a reduced to an increased risk of autism (RR 0.21, 2.59).

#### Subgroup Analysis

See Supplementary Figures S2–S6. In high-quality observational studies there was a 23% reduced risk of autism associated with maternal multivitamin use, and heterogeneity was reduced from considerable to substantial (RR 0.77, 95% CI: 0.62, 0.96, I^2^ = 62.4%). No association was observed in low-quality studies (RR 0.78, 95% CI: 0.33, 1.86, I^2^ = 97.9%), and there were no between-subgroup differences detected Q = 0.00, *p* = 0.98). Some evidence of association was observed in prospective studies (RR 0.69, 95% CI: 0.48, 1.00, I^2^ = 95.9%), whilst no association was evident in the retrospective studies (RR 0.93, 95% CI: 0.41, 2.11, I^2^ = 81.7%). There were no differences between subgroups for study design (Q = 0.42, *p* = 0.51). Subgroup analysis of regions reduced heterogeneity from “considerable” in Nordic (RR 0.87, 95% CI: 0.72, 1.06, I^2^ = 76.8%) and American (RR 0.87, 95% CI: 0.5, 2.15, I^2^ = 84.5 %) studies. Asian countries had the largest effect estimate, but there was considerable heterogeneity (RR 0.56, 95% CI: 0.26, 1.23, I^2^ = 97.1%). No differences between subgroups were detected (Q = 1.14, *p* = 0.57). Subgroup analysis of the stage of pregnancy showed similar effect estimates in each group (early pregnancy: RR 0.76, 95% CI: 0.58, 0.99, I^2^ = 79.8%; any stage of pregnancy: RR 0.78, 95% CI: 0.40, 1.53, I^2^ = 96.6%). There were no between-subgroup differences (Q = 0.01, *p* = 0.93). Regions without mandatory fortification produced a stronger reduction in the risk of autism (RR 0.71, CI 95%: 0.50, 1.02, I^2^ = 96%), compared to regions with mandatory fortification (RR 0.87, CI 95%: 0.35, 2.15, I^2^ = 84%). There were no between-subgroup differences detected (Q = 1.14, *p* = 0.57).

### 3.4. Causal Approaches

All studies [19,22,23,24,25,26,27,28,29,30] measured the association between prenatal nutrition and autism using conventional multivariate regression. Alternative causal approaches (one discordant sibling analysis, two negative controls studies, and one genetic interaction study) were infrequently applied (Table 4) and were generally used as a secondary analysis to conventional multivariate regression. A detailed summary of the key sources of bias in each approach can be read in Supplementary Table S6.

## 4. Discussion

Prenatal multivitamin supplements were not robustly associated with autism in the overall meta-analysis. However, a reduced risk of autism was observed in high-quality studies, prospective studies, early pregnancy and following the removal of an outlier. In contrast with previous meta-analyses, we did not observe any strong evidence of association in our main results (using all studies) due to the selection of the Hartung–Knapp–Sidik–Jonkman estimator, rather than DerSimonian–Laird which underestimates uncertainty and was applied in previous meta-analysis [4,5,31]. We also identified an additional two studies [21,27]. However, although some associations were identified, based on GRADE the degree of certainty was very low owing to the inherent risk of bias in observational study designs, considerable heterogeneity, and unexplained inconsistency in the direction of effect. As GRADE does not easily incorporate alternative causal approaches [32], we structured the discussion first to discuss the limitations identified through the application of GRADE. Second, we evaluated whether the alternative causal methods had been of value to the interpretation of causality.

Regional variation in baseline nutritional status and genotype [5,33] may be major contributors to both heterogeneity and inconsistency in the direction of the effect. Nutrients confer a benefit to health until physiological requirements are satisfied; thereafter, we observe a plateau effect, and toxicity or deficiency occurs when intakes are extreme [34]. To illustrate this potential U-shaped relationship, we considered baseline folate status. Studies in this review from Nordic countries generally showed effect estimates closest to the null, except Norway [24,25]. The Nordic associations correlated with rates of plasma folate deficiency/insufficiency, which were reported to be 0.7% in Denmark [35], 4% in Sweden [36], but 24.9% in a subsample of the Norwegian mother, father and child cohort [37]. However, the comparisons between supplement use and plasma folate levels were drawn from different populations, and population heterogeneity and confounding may have caused variance at the individual level. Future studies should consider the response to nutritional supplements in relation to baseline nutritional status.

Furthermore, from the reviewed studies, only the U.S. implements mandatory fortification of diet with folic acid and so has a high baseline folic acid intake. Less than 1% of its population has had deficient plasma folate levels since the introduction of mandatory fortification in 1998 [38], but this may have reduced the benefits from supplements since physiological requirements are already met. However, a plateau effect was not observed. Instead, two U.S. studies found a reduced risk of autism associated with multivitamin supplements [23,28], and two studies observed an increased risk of autism [20,29]. There is much uncertainty and debate as to whether toxicity could occur through the combined effects of mandatory fortification and supplementation with folic acid. [39]. Wiens and DeSoto (2017) argued that excessive intake saturates metabolic pathways, leading to an accumulation of unmetabolized folic acid which may cause autism [39]. Furthermore, folic acid is absorbed more readily than the folate form and is used in supplements and fortified food which the authors argued further exacerbates excessive intake. However, other research groups found inconclusive evidence to support such claims, even though limitations in available evidence were acknowledged [40].

The two studies in this review that observed a reduced risk of autism had folic acid intakes well in excess of the recommended 400 ug/day [23,28], yet they focused on early pregnancy when, it is speculated, folic acid requirements and tolerance to high doses is greater. An alternative explanation for the increased risk of autism is “birth order bias” [41]. Autism and supplement use can be positively correlated independently, as supplement use is usually greater in first pregnancies, and families affected by autism have fewer children. Indeed, an increased risk of autism in Moser et al. was attenuated to the null with restrictions to first-born boys [27].

An additional source of heterogeneity is variation in the stage of pregnancy when the multivitamin supplement was taken. In early pregnancy it was associated with a lower risk of autism and lower heterogeneity. Periconception is an established critical period in which FA can reduce the incidence of neural tube defects by up to 70% [42]. Furthermore, FA in periconception may reduce the risk of low birth weight, small gestational age size, stillbirth, neonatal mortality, preeclampsia and miscarriage [42].

Lastly, autism is a heterogeneous condition and specific features might have differential causal pathways [43], which may contribute to varied results. All studies that stratified by severity, observed a greater magnitude of association with more severe forms of autism [21,22,24,26] as defined by minimal verbal status at age 3 [24], low intelligence quotient [22,26], or high autism symptom severity [21].

### 4.1. Alternative Causal Approaches

#### 4.1.1. Multivariate Regression

Alternative causal approaches were infrequently applied, and the application was often not robust; therefore, our ability to triangulate the findings was limited. All studies used multivariate regression, a key assumption of which is no residual confounding [7]. However, there was such a risk in this review as studies were adjusted for many, but seldom all, key confounders. The studies commonly adjusted formaternal age, physical or mental health, socioeconomic status, parity, planned pregnancy, pre-pregnancy BMI, and health behaviour. Due to the risk of residual confounding we attempted to triangulate the results from the multivariate regression with alternative approaches that have different sources of bias. However, only the gene-nutrient interaction analysis provided useful results.

#### 4.1.2. Gene-Nutrient Interaction

In Schmidt et al., an association between folate use and autism risk was only observed if the mother/child had the methylenetetrahydrofolate reductase (MTHFR) 677 C > T genotype, which is unlikely owing to confounding by socioeconomic and lifestyle characteristics [28]. The MTHFR 677 C > T genotype encodes for a less-efficient enzyme to metabolise folate. Hence, larger folate doses may be necessary to overcome inefficient enzymatic function [28]. Although these findings are yet to be replicated in larger samples, there is consistency in the wider literature. A recent meta-analysis identified an 86% increased risk of autism associated with the less-efficient genotype (TT genotype verses CC genotype: OR 1.86, 95% CI: 1.12, 2.18) [44]. Furthermore, this association was not evident in countries that had a higher intake of folic acid secondary to mandatory folic acid fortification. This may imply that a genotype influences the response to supplements or mandatory folic acid fortification.

#### 4.1.3. Discordant Sibling Analysis

Discordant sibling analyses may overcome shared unmeasured confounding despite several key methodological considerations. In DeVilbiss et al., the reduced risk of autism observed in their main analysis was attenuated by the discordant sibling analysis [22]. Sibling comparison studies are a quasi-experimental study design intended to remove shared familial confounding by matching siblings discordant for the outcome. However, this includes shared genetic risk factors [7], and siblings share, on average, 50% of their genetic material. As indicated in Schmidt et al.’s study, it may be the combination of MTHFR 677 C > T genotype and no folic acid supplements that led to an increased risk of autism. Thus, this sibling comparison may have adjusted for a causal component. Furthermore, there is a high type II error rate as only siblings discordant for the exposure and outcome contribute to the effect estimate in conditional logistic regression models [45]. DeVilbiss et al.’s discordant sibling analysis may be underpowered because the confidence intervals were wider even though the point estimate was consistent with the main analysis. Furthermore, random error can be amplified in discordant sibling analysis and bias towards the null. Conversely, time-varying confounders should be adjusted as matching on siblings does not account for this [45]. Thus, the null association observed here should be interpreted with caution as it could reflect a type II error or adjustment for a causal component, MTHFR genotype.

#### 4.1.4. Negative Control

In two studies, negative controls were applied and acted as mock exposures that indicate the presence of bias without relying on the assumption of no unmeasured confounding [46]. Instead, they depend on the assumption that confounding, and sometimes other biases, were similar in exposure and negative control analyses. A further assumption was that the negative control had no plausible relationship with the outcome. Therefore, the exposure–outcome relationship could be distinguished from bias by comparing the strength of the association with the negative control analysis [7], but the assumptions were empirically untestable. Thus, we were limited to a subjective interpretation based on subject knowledge [46] which is presented here.

Suren et al. measured an association with multivitamin supplements, but not fish oils [24] even though they are a rich source of polyunsaturated fatty acids, especially omega-3s, and cod liver oil is a rich source of vitamin D, both of which are associated with positive neurodevelopmental outcomes [47,48]. Conversely, cod liver oil is a rich source of vitamin A which is potentially teratogenic and can harm foetal development [49]. Thus, this negative control violates the assumption of no plausible relationship with the outcome, which confused the interpretation. Similarly, Levine et al.’s negative control may have had a relationship with the exposure. The authors compared mutually exclusive groups for multivitamins: two years prior to pregnancy, two years prior to pregnancy and during pregnancy, and during pregnancy only. All groups were associated with a reduced risk of autism, yet the association was strongest and most similar for “two years prior to pregnancy” and “two years prior to pregnancy and during pregnancy”. No “wash-out” period was used, so two years prior to pregnancy may have included the potentially critical period, preconception. Furthermore, the assumption of similar bias may not be met either since women who discontinue supplements during pregnancy may have different characteristics to women who adhere to health advise and take the supplements during pregnancy. Thus, we lacked confidence in this negative control.

#### 4.1.5. Triangulation

Collectively, triangulation as a strategy to further causal interpretation was limited, many due to infrequent use and limitations in applying alternative approaches. The multivariate regression and gene-nutrient interaction [24] findings suggest there could have been a causal association, and we feel this warrants further investigation. However, within the context of this review, the discordant sibling analysis [22] and negative control analyses [24,26] were of limited utility. The former needs to be conducted on larger samples and adjusted for a range of time-varying confounders, and the choice of the latter should be given careful consideration.

### 4.2. Strengths and Limitations

This review has several strengths. Numerous steps to reduce bias were taken, such as searching grey literature sources, applying GRADE guidelines, calculating prediction intervals and using the Hartung–Knapp–Sidik–Jonkman estimator. However, the strongest advance was a formal narrative synthesis of the range of causal approaches. As an explicit approach, it provided transparent evidence of the approaches applied, their findings and outlined areas for future studies. Nonetheless, there were weaknesses, mainly the heterogeneity and inconsistency observed across studies and the low study numbers. The prediction intervals indicated if there had been an effect, it could have ranged from beneficial to null or even harmful in individual settings.

## 5. Conclusions

At present, the evidence is inconclusive, and we are unable to confirm a causal association between prenatal multivitamin supplement use and autism in offspring. Future studies should improve the study design and data analyses through adequately powered prospective birth cohorts. The measurement of nutritional supplements could be improved through reporting their nutrient composition, dose, compliance and duration and timing of use, which are all known to affect biological responses but were rarely considered in the studies we reviewed. Furthermore, there should be greater consideration of the complexity of nutrition by modelling U-shaped relationships and considering how the response to nutrients is altered by variations in baseline requirements and whether it is affected by recent nutrient intake, changes to physiological demand in early pregnancy, or genetic variation. Lastly, we recommend the application of alternative approaches within a triangulation framework to gauge causality better.

## Figures and Tables

**Table 1:** Search strategy for MEDLINE (OVID).

Search String	Search Terms
Population	(Pregnancy OR Fetal development OR Prenatal).af. OR Fetal development/OR “fetal development”.af. OR “fetal programming”.af OR “fetal programing”.af
Exposure	NUTRITIONAL PHYSIOLOGICAL PHENOMENA OR Prenatal Nutritional Physiological Phenomena/OR Maternal nutritional physiological phenomena/OR DIET OR nutri* OR vitamin OR diet* OR mineral OR Nutritional Status

An asterisk (*) is used as truncation in Medline and allows any word ending.

**Table 2:** Summary of GRADE evaluation.

Study	Cases/Total Sample	RR	95% CI	Quality of Evidence (GRADE)
7 cohort/5 case-control	8761/1,025,534	0.74	0.53, 1.04	very low

**Table 3:** Study characteristics and results from individual studies [19,20,21,22,23,24,25,26,27,28,29,30].

Author/Study Design	Country/Cohort/Sample	Exposure Measure (S)	Outcome Measure	Covariates ^a^	Results Effect Size Estimate and 95% CI	
Desoto and Hitlan, 2012 [29]/case-control	USA 256 cases and 752 controls, age range 6–12 years	Self-reported FA or multivitamin supplement use obtained from health records	ICD-9	Child; anaemia, pica, sex, birth weight, birth order, year of birth, breast feeding, sex and FA interaction Maternal; age, poverty ratio, adequacy of prenatal care, cholesterol screen, pap smear, prenatal alcohol use, prenatal viral infections, lead exposure	Reference, no supplement use	
					FA/multivitamin	
					HR 2.34	1.14, 4.82

DeVilbiss et al., 2017 [22]/population-based cohort and sibling case control	Sweden/Stockholm youth cohort 273,107/1064 cases age range 4–15 years	Self-reported multivitamin supplement use recorded by midwife at first booking	DSM-IV or ICD-10	Child; sex, birth year, and years of residence in Stockholm County Maternal; country of birth, maternal education, disposable family income, age, parity, smoking, BMI at first antenatal visit, neurologic or psychiatric conditions before the child’s birth (anxiety disorders, autism, bipolar disorder, depression, intellectual disability, non-affective psychosis, stress related disorders, epilepsy), antiepileptics and antidepressants medication Sibling analysis child sex and birth year, maternal parity	Reference, no supplement use	
					Multivitamin	
					OR 0.89	0.82, 0.97
					Sibling analysis, multivitamin	
					OR 0.95	0.81, 1.13
					Propensity score matching	
					OR 0.86	0.78, 0.95

Levine et al., 2018 [26]/nested case-control	Israel/Meuhedet population based register 572 cases and 45,300 controls average age 10 years (SD 1.4)	Pharmacy records, Multivitamins and/or FA supplements	ICD-9	Child; sex, birth year Maternal; parity, socioeconomic status (high vs low), psychiatric diagnosis by childbirth, age Paternal psychiatric diagnosis, age	Reference, no supplement use	
					FA/multivitamins	
					RR 0.27	0.22, 0.33
					Negative control	
					Reference, no supplement use	
					Multivitamin use 2 years prior to pregnancy	
					0.12	0.07, 0.20

Li et al., 2018 [30]/case control	China/Autism clinical and environmental database 354 cases and 374 controls, average age 4.5 years	Self-reported FA supplements	DSM-IV-TR	Child; age, premature birth, gender Maternal; pre-pregnancy BMI preconception and predelivery, mode of delivery Parental; age, education Dietary patterns were additionally adjusted for other dietary patterns FA and calcium supplements: were additionally adjusted for other supplements.	Reference, no supplement use	
					FA	
					OR 0.64	0.41, 1.00

Moser et al., 2019 [27]/nested case-control	Israeli/Maccabi Healthcare Services 2009 cases and 19,886 controls	Dispensing records for FA with or without multivitamins supplements	DSM, version unreported	Child: sex, birth year, birth order Maternal: age, region of residence, poverty index, number of physician visits, diabetes mellitus, hypertension, cardiovascular disease, cancer, subfertility, epilepsy, antifolate medication (proguanil, methotrexate, sulfasalazine, sulphamethoxazole and trimethoprim, pyrimethamine, valproate, carbamazepine, phenytoin and phenobarbital) Final model adjusted for; maternal age, subfertility, number of physician visits, birth order, parity	Reference, ≤0.2 mg/day	
					FA supplement	
					0.2–<0.4 mg/day	
					OR 1.15	0.98, 1.24
					0.4–<1 mg/day	
					OR 1.10	0.98, 1.24
					1 < 3 mg/day	
					OR 1.14	0.98, 1.34
					≥3 mg/day	
					OR 1.01	0.60, 1.70

Raghavan et al., 2018 [20]/prospective cohort	USA/Boston Medical Centre 1257/86 cases, aged up to 9 year	Self-reported maternal multivitamin supplement use	ICD-9	Child; sex, gestational age birth year Maternal; homocysteine, race, age, smoking status, diabetes, reduction, parity, MTHFR 677 genotype, BMI	Reference, multivitamins 2–5 times/week	
					First trimester ≤ 2/week	
					HR 3.4	1.6, 7.2
					>5/week	
					HR 2.3	1.2, 3.9
					Second trimester ≤ 2/week	
					HR 3.8	1.8, 8.0
					>5/week	
					HR 2.1	1.2, 3.6
					Third trimester	
					≤2/week	
					HR 3.5	1.7, 7.4
					>5/week	
					HR 2.1	1.2, 3.6
Schmidt et al., 2019 [23]/prospective cohort	USA/Markers of Autism Risk in Babies: Learning Early Signs (MARBLES) 332/55 cases, average age 36.5 months	Self-reported multivitamin supplement use obtained through three telephone interviews	Autism Diagnostic Observation Schedule and DSM-5	Child; birthplace, sex, year of birth Maternal; education, age, pre-pregnancy BMI, planned pregnancy, race/ethnicity, home ownership, insurance delivery type. Paternal; age Covariates in final model, maternal characteristics (education, age, insurance delivery type) and child characteristics (race)	Reference, no supplement use	
					Multivitamin	
					RR 0.50	


Schmidt et al 2012 [28]/case control	USA/Childhood autism risks from genetics and environment (CHARGE) 429 cases and 278 controls age range 24–60 months	Self-reported obtained FA intake based on supplements, including multivitamin supplements, and fortified breakfast cereals, shakes and bars obtained via telephone interview	Health records and Autism Diagnostic Interview–Revised and the Autism Diagnostic Observation Schedule–Generic	Child; birth year, sex, race Maternal; race, age, education, pre-pregnancy BMI, birthplace, residing with a smoker, smoking status, alcohol consumption, other nutrients intakes (calcium, iron, vitamin A, B_6_, B_12_, C, D and E) Preeclampsia, type of delivery, vaginal bleeding during pregnancy, induced labour Paternal: age Covariates in final model; childbirth year and maternal education	Reference, no supplement use	
					All strata combined	
					OR 0.62	0.42, 0.92
					Strata by C/T genotypes	
					maternal CC	
					OR 1.20	0.61, 2.34
					maternal CT/TT	
					OR 0.46	0.25, 0.85
					child CC	
					OR 1.15	0.55, 2.38
					child CT/TT	
					OR 0.48	0.27, 0.88
					both mother & child	
					OR 1.29	0.54, 3.10
					either mother or child CT/TT	
					OR 0.49	0.16, 1.50
					both mother and child CT/TT	
					OR 0.30	0.10, 0.90
Suren et al., 2013 [24]/prospective cohort	Norway/Norwegian mother, father and child cohort (MoBa) 85,176/114 cases, mean age of 6.4 years	Self-reported questionnaire responses, multivitamin and mineral supplement, FA supplement	DSM-IV or ICD-10	Child; birth year Maternal; planned pregnancy, smoking, BMI, parity, weight gain at 18 and 30 weeks Parental; education, age Covariates retained in final model; birth year, parity and maternal education	Reference, no supplement use	
					FA/multivitamins	
					OR 0.61	0.41, 0.90
					Negative control	
					Reference, no fish oils supplements	
					Fish oil supplements	
					OR 1.29	0.88, 1.89
Strom et al., 2017 [19]/prospective cohort	Denmark/Danish National Birth Cohort 87,210/1234 cases, age range 11–17 years	Self-reported folate/FA, vitamin and mineral supplement use, reported during GP interview	ICD-10	Child; sex Maternal; age, parity, smoking, education, socioeconomic status (based on occupation and education), planned pregnancy, pre-pregnancy BMI Paternal; age	Reference, no supplement use	
					FA/multivitamins	
					HR 1.06	0.94, 1.19

Tan et al., 2020 [21]/case- control	China 416 cases and 201 control mean age 4.68 years cases, 4.47 years controls	Self-reported FA or micronutrient supplements	DSM-5	Child; age, sex, gestational age, birth weight Maternal; residence (rural/urban), labour mode Paternal; age Household; income	Reference, supplement use	
					No FA supplements	
					1.91	1.24, 2.93
					No micronutrient supplements	
					1.72	1.20, 2.47

Nilsen et al., 2013 [25]/population based cohort	Norway/Norwegian registry 507,856/2072 cases, mean age 7 years	Self-reported FA intake obtained via maternal health records	ICD-10	Child; birth year Maternal; age, marital status, parity, hospital size Paternal; age	Reference, no supplement use	
					FA	
					OR 0.86	0.78, 0.95


^a^ All covariates initially considered relevant by the authors are listed as well as the final selection. Initialisms: BMI, Body Mass Index; DSM, Diagnostic and Statistical Manual of Mental Disorders; DSM-IV-TR, Diagnostic and Statistical Manual of Mental Disorders Text Revision; FA, folic acid; GDM, gestational diabetes mellitus; GWG, gestational weight gain; ICD, International Classification of Disease; SD standard deviation; MTHFR 677, Methlyenetetrahydrofolate reductase 677 (genotype); CT/TT/CC, genotype variants; Q, Quintle; SNP, single-nucleotide polymorphism.

**Table 4:** Summary of causal approaches which demonstrates multivariate regression was commonly applied whilst all other approaches were infrequently used.

Study	Design	Sample Size Cases/Controls or Children (Cases)	Multivariate Regression/GEE	Causal Diagram	Propensity Score	Sibling Study	Negative Control	Genetic Studies
Folic acid/multivitamins								
Desoto and Hitlan, 2012 (taking supplements)	Case-control	256/752	▼					
DeVilbiss et al., 2017 (taking supplements)	Cohort	273,107 (1064)	▲		▲	Δ		
Levine et al., 2018 (taking supplements)	Cohort	45,300 (572)	▲				▲ ^b^	
Li et al., 2018 (taking supplements)	Case-control	354/374	▲					
Moser et al., 2019 (taking supplements)	Case-control	2009/19,886	Δ					
Nilsen et al., 2013 (taking supplements)	Cohort	507,856 (2072)	▲					
Raghavan et al., 2017 ^a^ (taking supplements < twice/week or taking supplements > five times/week)	Cohort	1257 (86)	▼					
Schmidt et al., 2019 (taking supplements)	Cohort	241 (55)	▲					
Schmidt et al., 2012 (taking supplements)	Case-control	429/278	▲					▲ ^c^
Suren et al., 2013 (taking supplements)	Cohort	85,176 (114)	▲				∇ ^d^	
Strom et al., 2017 (taking supplements)	Cohort	87,210 (1234)	∇					
Tan et al., 2020 (not taking supplements)	Case-control	416/201	▲					

Effect direction: ▲ indicates a positive health impact, ▼ indicates a negative health impact. Significance association indicated with a black arrow, no association is indicated with an unshaded arrow (Δ, ∇); The nutrient source is supplements/fortified food (reference group: no/low intake); ^a^ Raghavan et al. reference group is 3–5/week compared to low and high supplements intakes; ^b^ negative control was two years pre-pregnancy and had a stronger association with autism; ^c^ Beneficial effects of folic acid supplements were only detected if the child or mother had at least one T allele which reduces the efficiency of folate metabolism; ^d^ negative control was fish oils, which showed no association with autism.

## Data Availability

Available on request.

## Supplementary Materials

The following are available online at https://www.mdpi.com/article/10.3390/nu13082558/s1, Figure S1: Multivitamins: funnel plot, Figure S2: Multivitamin subgroup analysis by study quality, Figure S3: Multivitamin subgroup analysis by study design, Figure S4: Multivitamin subgroup analysis by region, Figure S5: Multivitamins subgroup analysis by stage of pregnancy, Figure S6: Multivitamins subgroup analysis by mandatory fortification. Table S1: Preferred Reporting Items for Systematic Reviews and Meta-Analyses, Table S2: Quality assessment using the Newcastle Ottawa Scale: case-control, Table S3: Quality assessment using the Newcastle Ottawa Scale: cohort, Table S4: GRADE evidence profile, Table S5: Optimal information size, Table S6: Summary of the key sources of bias for each causal approach, Table S7: Influential study analysis.
