# Supplementary material for: Prenatal Vitamins and the Risk of Offspring Autism Spectrum Disorder: Systematic Review and Meta-Analysis
Source: Nutrients. 2021 Jul 26;13(8):2558. doi: 10.3390/nu13082558 (PMC8398897; doi:10.3390/nu13082558)
Supplement: Supplementary file 1 [file nutrients-13-02558-s001.zip › nutrients-1277270-supplementary.pdf]

Supplementary Table S1. Preferred Reporting Items for Systematic Reviews and Meta-Analyses

Section/topic	#	Checklist item	Reported on page #
TITLE			
Title	1	Identify the report as a systematic review, meta-analysis, or both.	pp 1. (title page/section 1)
ABSTRACT			
Structured summary	2	Provide a structured summary including, as applicable: background; objectives; data sources; study eligibility criteria, participants, and interventions; study appraisal and synthesis methods; results; limitations; conclusions and implications of key findings; systematic review registration number.	pp 2. (section 1)
INTRODUCTION			
Rationale	3	Describe the rationale for the review in the context of what is already known.	pp 1-2.
Objectives	4	Provide an explicit statement of questions being addressed with reference to participants, interventions, comparisons, outcomes, and study design (PICOS).	pp 2.
METHODS			
Protocol and registration	5	Indicate if a review protocol exists, if and where it can be accessed (e.g., Web address), and, if available, provide registration information including registration number.	pp 2.
Eligibility criteria	6	Specify study characteristics (e.g., PICOS, length of follow-up) and report characteristics (e.g., years considered, language, publication status) used as criteria for eligibility, giving rationale.	pp 3.
Information sources	7	Describe all information sources (e.g., databases with dates of coverage, contact with study authors to identify additional studies) in the search and date last searched.	pp 3.
Search	8	Present full electronic search strategy for at least one database, including any limits used, such that it could be repeated.	pp 3.
Study selection	9	State the process for selecting studies (i.e., screening, eligibility, included in systematic review, and, if applicable, included in the meta-analysis).	pp 3-4.
Data collection process	10	Describe method of data extraction from reports (e.g., piloted forms, independently, in duplicate) and any processes for obtaining and confirming data from investigators.	pp 3.

Data items	11	List and define all variables for which data were sought (e.g., PICOS, funding sources) and any assumptions and simplifications made.	pp 3.
Risk of bias in individual studies	12	Describe methods used for assessing risk of bias of individual studies (including specification of whether this was done at the study or outcome level), and how this information is to be used in any data synthesis.	pp 3.
Summary measures	13	State the principal summary measures (e.g., risk ratio, difference in means).	pp 3-4.
Synthesis of results	14	Describe the methods of handling data and combining results of studies, if done, including measures of consistency (e.g., I^2) for each meta-analysis.	pp 3-4.
Risk of bias across studies	15	Specify any assessment of risk of bias that may affect the cumulative evidence (e.g., publication bias, selective reporting within studies).	pp 3
Additional analyses	16	Describe methods of additional analyses (e.g., sensitivity or subgroup analyses, meta-regression), if done, indicating which were pre-specified.	pp 4.
RESULTS			
Study selection	17	Give numbers of studies screened, assessed for eligibility, and included in the review, with reasons for exclusions at each stage, ideally with a flow diagram.	pp 4
Study characteristics	18	For each study, present characteristics for which data were extracted (e.g., study size, PICOS, follow-up period) and provide the citations.	pp 6-11.
Risk of bias within studies	19	Present data on risk of bias of each study and, if available, any outcome level assessment (see item 12).	pp. 5
Results of individual studies	20	For all outcomes considered (benefits or harms), present, for each study: (a) simple summary data for each intervention group (b) effect estimates and confidence intervals, ideally with a forest plot.	pp 12.
Synthesis of results	21	Present results of each meta-analysis done, including confidence intervals and measures of consistency.	pp 12-13
Risk of bias across studies	22	Present results of any assessment of risk of bias across studies (see Item 15).	pp. 5
Additional analysis	23	Give results of additional analyses, if done (e.g., sensitivity or subgroup analyses, meta-regression [see Item 16]).	pp 12-13
DISCUSSION			
Summary of evidence	24	Summarize the main findings including the strength of evidence for each main outcome; consider their relevance to	pp 16-19

		key groups (e.g., healthcare providers, users, and policy makers).	
Limitations	25	Discuss limitations at study and outcome level (e.g., risk of bias), and at review-level (e.g., incomplete retrieval of identified research, reporting bias).	pp 19
Conclusions	26	Provide a general interpretation of the results in the context of other evidence, and implications for future research.	pp 19
FUNDING			
Funding	27	Describe sources of funding for the systematic review and other support (e.g., supply of data); role of funders for the systematic review.	pp 19

Supplementary table S2 Quality assessment using the Newcastle Ottawa Scale: case-control. Scores ranged from 0-9, where 9 indicates the highest quality

Authors	Selection	Comparability	Exposure	Total
DeSoto & Hitlan 2013	2	1	1	4
Li et al 2018	2	1	1	4
Moser et al 2019	4	2	2	8
Schmidt et al 2012	3	2	2	7
Tan et al 2020	2	1	1	4

Supplementary table S3. Quality assessment using the Newcastle Ottawa Scale: cohort Scores ranged from 0-9, where 9 indicates the highest quality

Authors	Selection	Comparability	Outcome	Total
DeVilbiss et al 2017	3	2	3	8
Levine et al 2018	3	2	1	6
Raghavan et al 2017	3	2	3	8
Schmidt et al 2019	3	2	2	7
Strom et al 2017	2	1	2	5
Suren et al 2013	3	2	3	8
Nilsen et al 2013	3	1	3	7

Supplementary table S4 GRADE evidence profile

Quality assessment							Summary of findings		
Number of studies	Limitations	Inconsistency	Indirectness	Imprecision	Publication bias	Upgrade	Sample size cases/total sample	RR (95% CI)	Quality of evidence GRADE
12	no serious limitations	[a]serious inconsistency	no serious indirectness	no serious imprecision	no serious publication bias	not upgraded	8,761/1,025,534	0.74 (0.53, 1.04)	very low

[a] considerable heterogeneity only partially explained by study quality. Two study had harmful effects that were distinctly different from the body of evidence which was unexplained.

Supplementary table S5 Optimal information size

**RR**	20%	25%	30%
**Population 1**	0.01	0.01	0.01
**Population 2**	0.012	0.0125	0.013
**Sample size for each group**	42,693	27,937	19,827

based on 1% baseline risk, alpha = 0.05, power = .80

Supplementary table S6 Summary of the key sources of bias for each causal approach

**Causal approach**	**Generic description of causal approach and assumptions**	**Description of approach in included studies**	**Key sources and direction of bias in relation to the main assumptions of each approach for included studies**
Multivitamin			
Multivariate regression	Adjustment for confounders is achieved through statistical adjustment for multiple covariates. Key assumptions are no residual confounding from either poorly measured or unmeasured covariates; regression model is correctly specified.	1,025,534 children including 8,761 autism cases were synthesised, two studies were narratively synthesised only and 10 studies meta-analysed.	Generally good adjustment for the main known confounders except planned pregnancy which may exaggerate the magnitude of association.<br>Causes of autism are not fully known and so there remains potential for unobserved confounding that biases the association in either direction.<br>Inappropriate covariate selection was evident due to adjustment for mediators and/or conditions co-morbid with the outcome or exclusion of mediators in study selection criteria may bias towards the null or introduce error which biases in either direction.
Discordant sibling analysis	Compares sibling who are discordant for the exposure to adjust for shared familial confounding.<br>Key assumption is that time-varying confounding is minimised or adjusted, and there is minimal random error.	One sibling study [43] found an association in the general population multivariate regression which was attenuated in the discordant sibling analysis.<br>The study sample included 3066 mothers discordant for multivitamins supplement use and 7122 children discordant for autism.	The authors had a large sample size, but it was substantially smaller than that required based on our power calculation in Supplementary table 5 which may attenuate the association.<br>Time-varying covariates adjusted were child sex, birth year and parity. However, other variables such as maternal age and planned pregnancy were unadjusted. Maternal age increases the risk of autism and planned pregnancy may reduce the risk of autism.<br>The overall impact of unmeasured confounding is unclear.

Negative control	Negative controls have similar covariate structure to the exposure-outcome relationship but are not related to the outcome. Key assumption is the major sources of bias for the exposure and negative control are similar. They should be scaled similarly, especially when the negative control is the exposure taken at a less biologically plausible period.	One study [50] measured 85176 children, of which 114 had autism and found an association with multivitamin supplement use and not the negative control (fish oils supplements). Second study [46] measured 45,300 children and 572 cases and found stronger association between multivitamin supplements and autism 'two years prior to pregnancy' than 'during pregnancy', and a similar magnitude of association with 'pre-pregnancy and pregnancy'.	In the first study [50] fish oils may reduce the risk of autism and so have a relationship with autism, which could confuse the interpretation of the negative control. The second study found counter intuitive results and may reflect different sources of bias between the negative control and exposure, as well as the negative control having a relationship with autism [46]. Thus, their results are difficult to interpret.
Gene-nutrient interaction	Measures the interaction between genotype and nutrient Key assumption is no population stratification which occurs when groups of individuals have systematic differences in their genetic ancestry and the phenotype which may create a spurious association.	One study [31] measured 332 children and 55 cases and identified an interaction between folate supplements/fortified foods and the methylenetetrahydrofolate reductase 677 genotype. A reduced risk of autism in association with prenatal folic acid supplements was only identified if the mother or infant had the C>T allele which is associated with less efficient folate metabolism.	The main limitations are that the study has a small sample size and is yet to be replicated. We cannot be certain of the direction of bias this could confer. The authors tested for population stratification and reported no evidence of population stratification based on the expected Hardy-Weinberg equilibrium proportions ($P > 0.05$).

Discordant sibling analysis	as above	The first discordant sibling analysis [43] on 3066 children discordant for iron intakes and 7122 children discordant for autism, found no association in the general population or sibling analysis<br><br>The authors [40] of the discordant sibling analysis found an association between anaemia in earlier pregnancy (≤30 weeks gestation) and autism, in both the general population cohort and the discordant sibling analysis. The authors describe their sample size as 21 835 siblings, of which 6332 sibling had autism and were exposed to anaemia paired to neurotypical sibling unexposed to anaemia, and 1310 sibling pairs with the neurotypical sibling exposed to anaemia paired to the autistic sibling unexposed to anaemia.	In the first study [43] there was likely a lack of statistical power to detect an effect in the sibling analysis however, as we did not observe an association in the main analysis either, and so insufficient power alone is unlikely to explain the lack of association. Time-varying covariates adjusted for were sex, birth year and parity, however interpregnancy interval was not adjusted for.<br><br>In the second discordant sibling analysis [40] which measured anaemia showed shared familial confounding is unlikely to drive the association here. Time varying covariates adjusted were sex, birth year, and interpregnancy interval.

Table is adapted from Lawlor [8]. Limitations are discussed in the main body of the text.

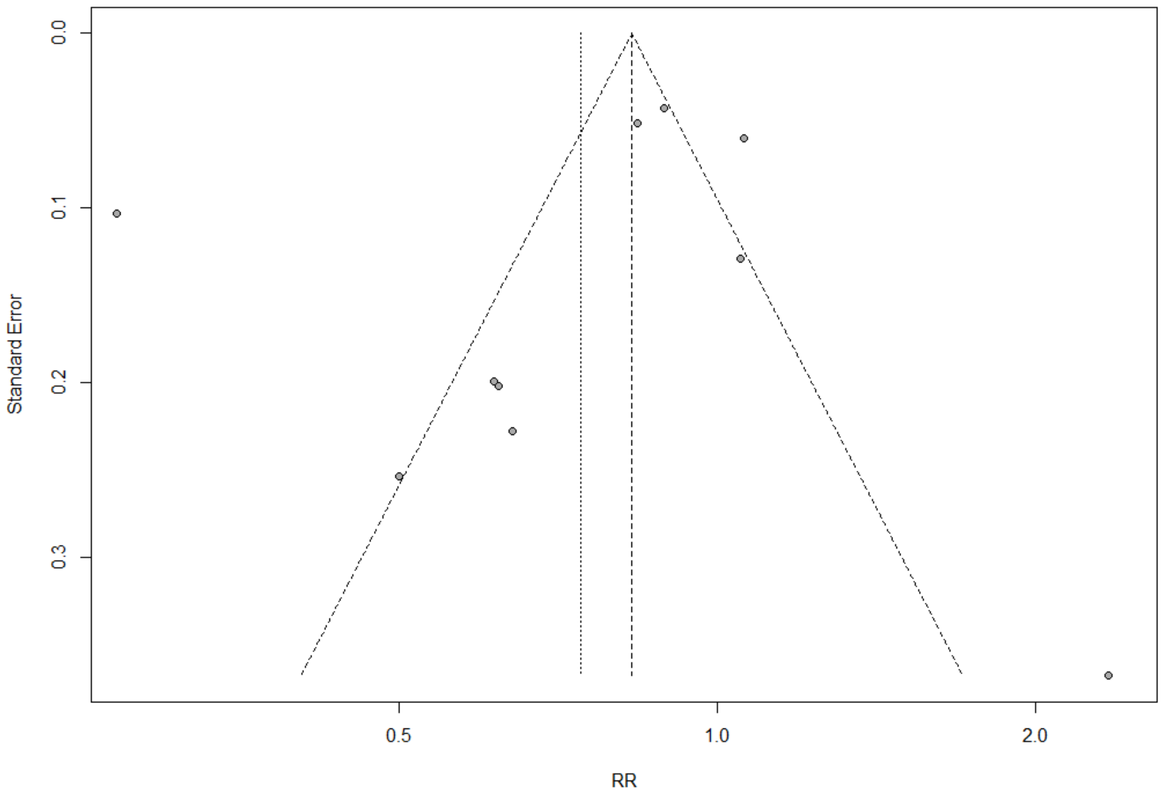

Supplementary figure S1. Multivitamins: funnel plot

Supplementary table S7 Influential study analysis

Author omitted	RR	95% CI	I²
Levine et al 2018	0.84	0.64, 1.10	75.1%
Schmidt et al 2019	0.77	0.54, 1.11	94.8%
Suren et al 2013	0.76	0.53, 1.10	94.9%
Schmidt et al 2012	0.76	0.52, 1.10	94.9%
Li et al 2018	0.76	0.52, 1.10	94.9%
Nilsen et al 2013	0.73	0.50, 1.07	94.9%
DeVilbiss et al 2017	0.73	0.50, 1.06	94.8%
Moser et al 2019	0.71	0.49, 1.03	94.8%
Strom et al 2018	0.71	0.49, 1.03	94.2%
DeSoto & Hitlan 2012	0.68	0.51, 0.91	94.7%
Summary estimate	0.74	0.53, 1.04	94.3%

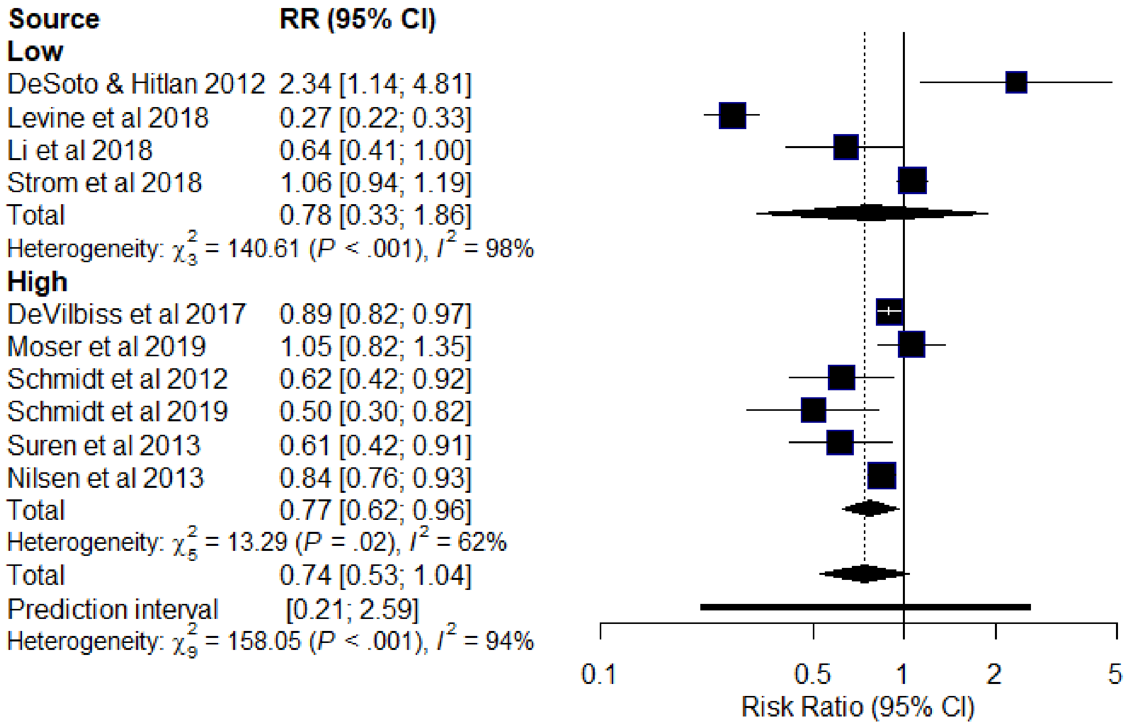

Supplementary figure S2. Multivitamin subgroup analysis by study quality

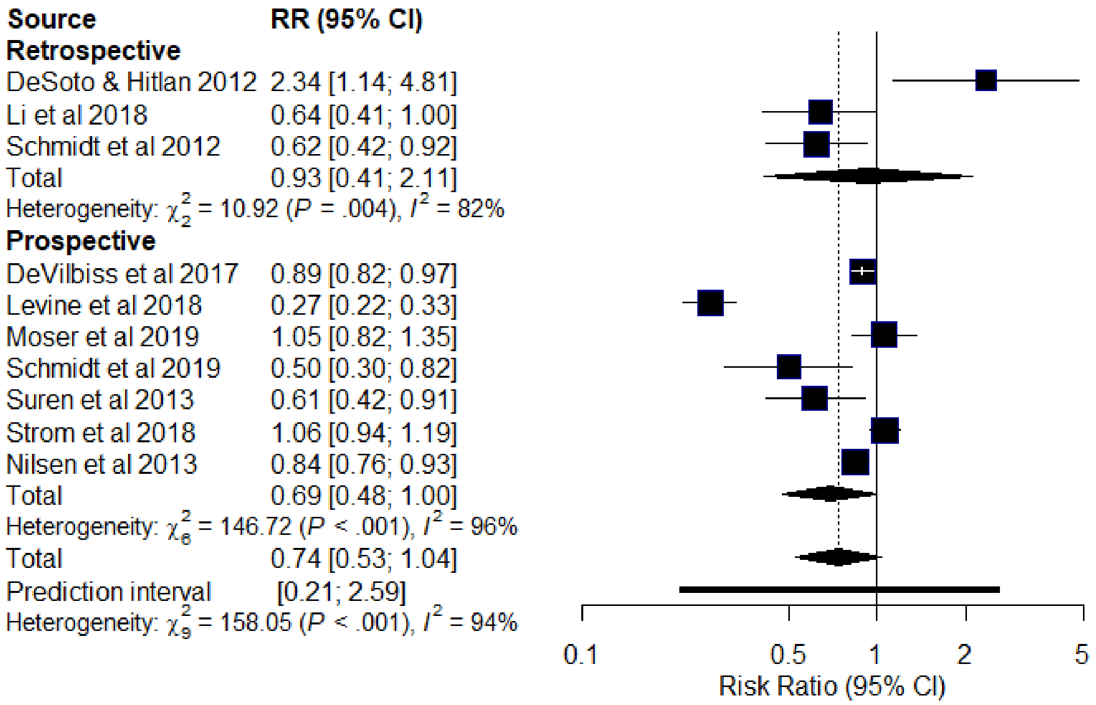

Supplementary figure S3. Multivitamin subgroup analysis by study design

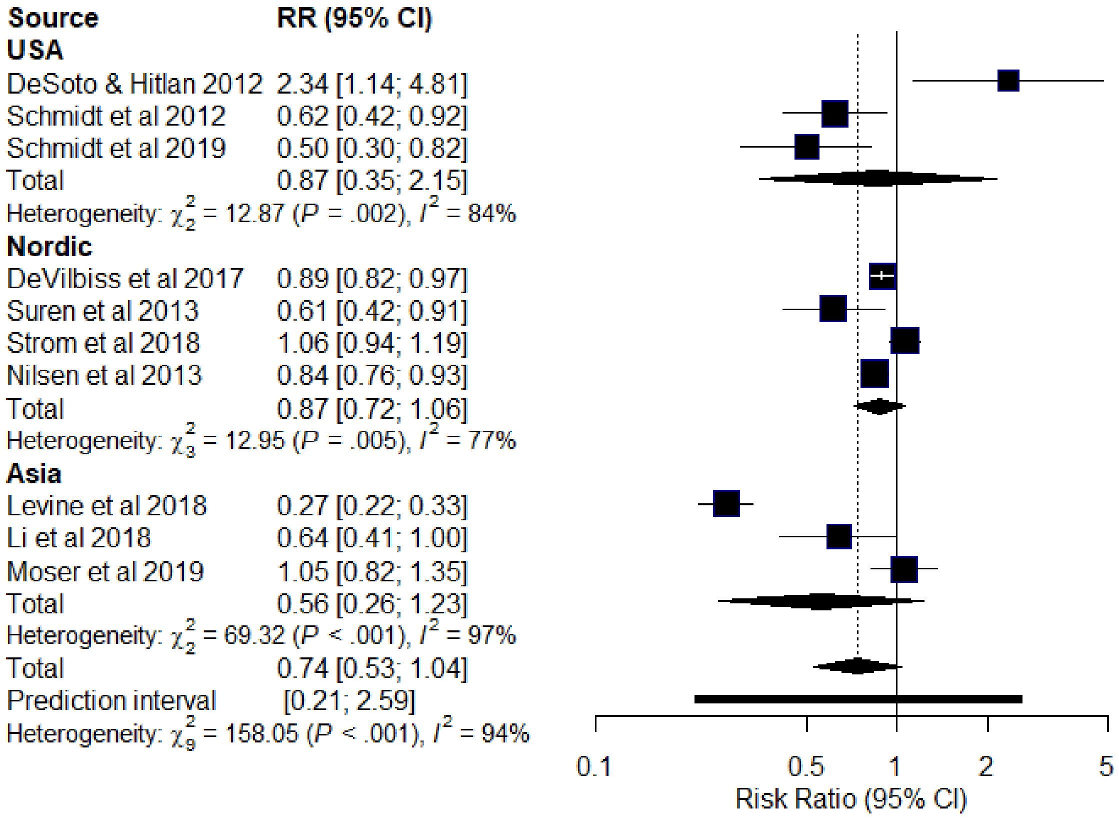

Supplementary figure S4. Multivitamin subgroup analysis by region

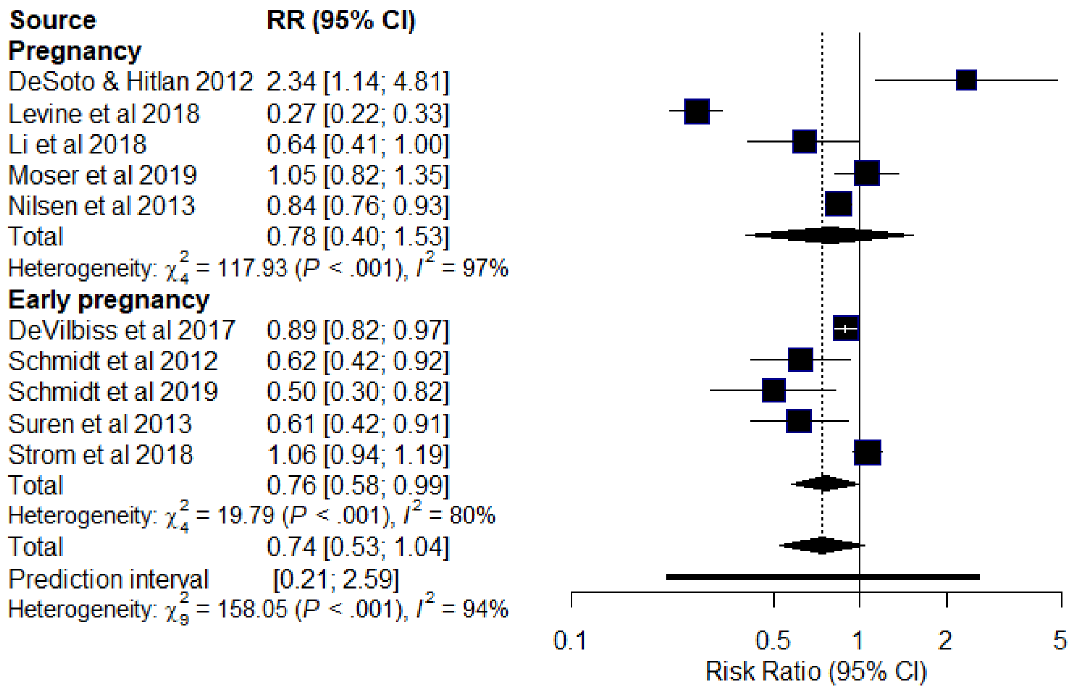

Supplementary figure S5. Multivitamins subgroup analysis by stage of pregnancy

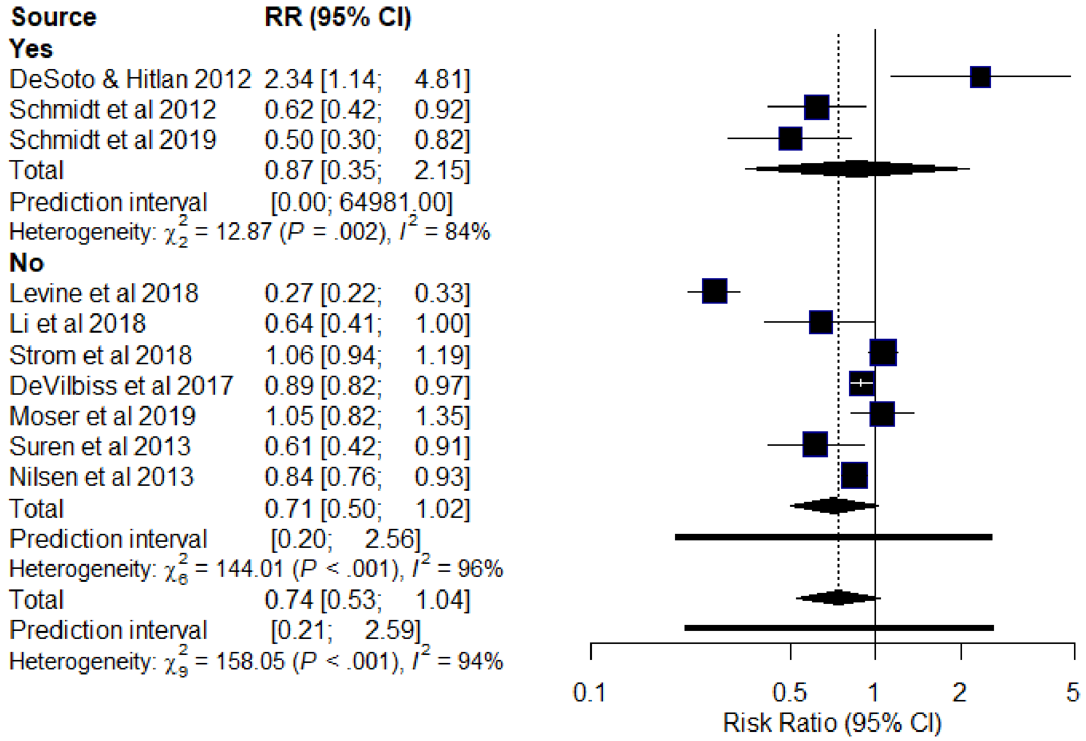

Supplementary figure S6. Multivitamins subgroup analysis by mandatory fortification. Yes, the country has mandatory fortification. No, the country does not have mandatory fortification
